# Accuracy of endoscopic diagnosis of *Helicobacter pylori* infection according to level of endoscopic experience and the effect of training

**DOI:** 10.1186/1471-230X-13-128

**Published:** 2013-08-15

**Authors:** Kazuhiro Watanabe, Naoyoshi Nagata, Takuro Shimbo, Ryo Nakashima, Etsuko Furuhata, Toshiyuki Sakurai, Naoki Akazawa, Chizu Yokoi, Masao Kobayakawa, Junichi Akiyama, Masashi Mizokami, Naomi Uemura

**Affiliations:** 1 Department of Gastroenterology and Hepatology, National Center for Global Health and Medicine, 1-21-1 Toyama, Shinjuku-ku, Tokyo 162-8655, Japan; 2 Department of Clinical Research and Informatics, National Center for Global Health and Medicine, 1-21-1 Toyama, Shinjuku-ku, Tokyo 162-8655, Japan; 3 Research Center for Hepatitis and Immunology, National Center for Global Health and Medicine, Kohnodai Hospital, 1-7-1 Kohnodai, Ichikawa City, Chiba 272-8516, Japan; 4 Department of Gastroenterology and Hepatology, National Center for Global Health and Medicine, Kohnodai Hospital, 1-7-1 Kohnodai, Ichikawa City, Chiba 272-8516, Japan

**Keywords:** Helicobacter pylori, Endoscopic training, Diagnostic yield, Endoscopic career level, Inter-observer agreement, Intra-observer agreement

## Abstract

**Background:**

Accurate prediction of *Helicobacter pylori* infection status on endoscopic images can contribute to early detection of gastric cancer, especially in Asia. We identified the diagnostic yield of endoscopy for *H. pylori* infection at various endoscopist career levels and the effect of two years of training on diagnostic yield.

**Methods:**

A total of 77 consecutive patients who underwent endoscopy were analyzed. *H. pylori* infection status was determined by histology, serology, and the urea breast test and categorized as *H. pylori-*uninfected, -infected, or -eradicated. Distinctive endoscopic findings were judged by six physicians at different career levels: beginner (<500 endoscopies), intermediate (1500–5000), and advanced (>5000). Diagnostic yield and inter- and intra-observer agreement on *H. pylori* infection status were evaluated. Values were compared between the two beginners after two years of training. The kappa (K) statistic was used to calculate agreement.

**Results:**

For all physicians, the diagnostic yield was 88.9% for *H. pylori*-uninfected, 62.1% for *H. pylori*-infected, and 55.8% for *H. pylori*-eradicated. Intra-observer agreement for *H. pylori* infection status was good (K > 0.6) for all physicians, while inter-observer agreement was lower (K = 0.46) for beginners than for intermediate and advanced (K > 0.6). For all physicians, good inter-observer agreement in endoscopic findings was seen for atrophic change (K = 0.69), regular arrangement of collecting venules (K = 0.63), and hemorrhage (K = 0.62). For beginners, the diagnostic yield of *H. pylori-*infected/eradicated status and inter-observer agreement of endoscopic findings were improved after two years of training.

**Conclusions:**

The diagnostic yield of endoscopic diagnosis was high for *H. pylori*-uninfected cases, but was low for *H. pylori*-eradicated cases. In beginners, daily training on endoscopic findings improved the low diagnostic yield.

## Background

Since the discovery of *Helicobacter pylori* in 1982 [1], the association between *H. pylori* infection and gastric cancer has been well established [2]. Moreover, recent studies have shown that eradication of *H. pylori* prevents development of metachronous gastric cancer [3,4]. However, gastric cancer can occur in not only *H. pylori-*infected patients, but also *H. pylori-*eradicated patients. Therefore, it is extremely important to determine *H. pylori* infection status (uninfected, infected, or eradicated) by regular screening endoscopy.

Among patients with *H. pylori*-infected gastric mucosa, atrophic change is considered to be a risk of gastric cancer [2]. In Asia, especially in Japan, severe atrophic gastritis is more common than in the West [5,6], and it is considered highly detectable on endoscopy in these regions [5]. Therefore, endoscopic visualization of *H. pylori* infection of the gastric mucosa is useful for the early detection of gastric cancer, and education of beginner endoscopists on this paradigm is becoming an important clinical issue. However, the value of endoscopic diagnosis of *H. pylori* infection status remains unclear [7-11].

In this paper, we identify the accuracy and reproducibility of endoscopic diagnosis of *H. pylori-*uninfected, -infected, and -eradicated status. Moreover, we compare scores of endoscopists with various levels of experience, and we identify the effect of two years of training on beginner endoscopists.

## Methods

### Subjects

A total of 148 consecutive dyspeptic patients who had undergone upper gastrointestinal endoscopy and who were diagnosed strictly for *H. pylori* infection at the National Center for Global Health and Medicine (NCGM) between December 2008 and April 2009 were selected from an endoscopic electronic database. Exclusion criteria included the use of non-steroidal anti-inflammatory drugs (NSAIDs), anti-thrombogenic drugs, and proton pump inhibitor and patients with a history of gastric surgery, hemorrhagic disease, liver cirrhosis, end-stage renal disease requiring dialysis, severe heart failure with any symptoms, and early or advanced gastric cancer, because these conditions can affect the mucosal appearance of the stomach [12-15]. After exclusion, 77 patients were selected for analysis.

Written informed consent was obtained from all participants in accordance with the Declaration of Helsinki and its subsequent revision. The study protocol was approved by the Ethics Committee of the NCGM (approval No. 811).

### Gold standard for diagnosis of H. pylori infection status

*H. pylori* infection was evaluated by the presence of serum immunoglobulin G antibody against *H. pylori* (HM-CAP, Enteric Products, Westbury, NY), a ^13^C urea breath test (UBT; with a cut-off value of 2.5‰; Ubit, Otsuka Pharmaceuticals, Tokyo, Japan), and histological examination with toluidine blue staining. For histological evaluation, three endoscopic biopsy specimens were taken from the greater curvature of the upper gastric body, angulus, and antrum.

Subjects with a history of *H. pylori* eradication who were confirmed negative by histologic examination of gastric biopsy specimens and a negative ^13^C-UBT were defined as eradicated patients. Subjects without a history of *H. pylori* eradication who were confirmed negative based on the results of all three methods were defined as uninfected patients. The remaining subjects in whom neither status was confirmed were defined as infected patients.

### Endoscopic assessment of H. pylori infection status

All endoscopies were performed by well-trained endoscopists using a high resolution videoendoscope (GIF-260H, Olympus Medical Systems, Tokyo, Japan) with a pre-endoscopic oral solution containing dimethylpolysiloxane (Balgin Antifoaming Oral Solution 2%, Kaigen Co., Ltd., Osaka, Japan).

**Figure 1:**
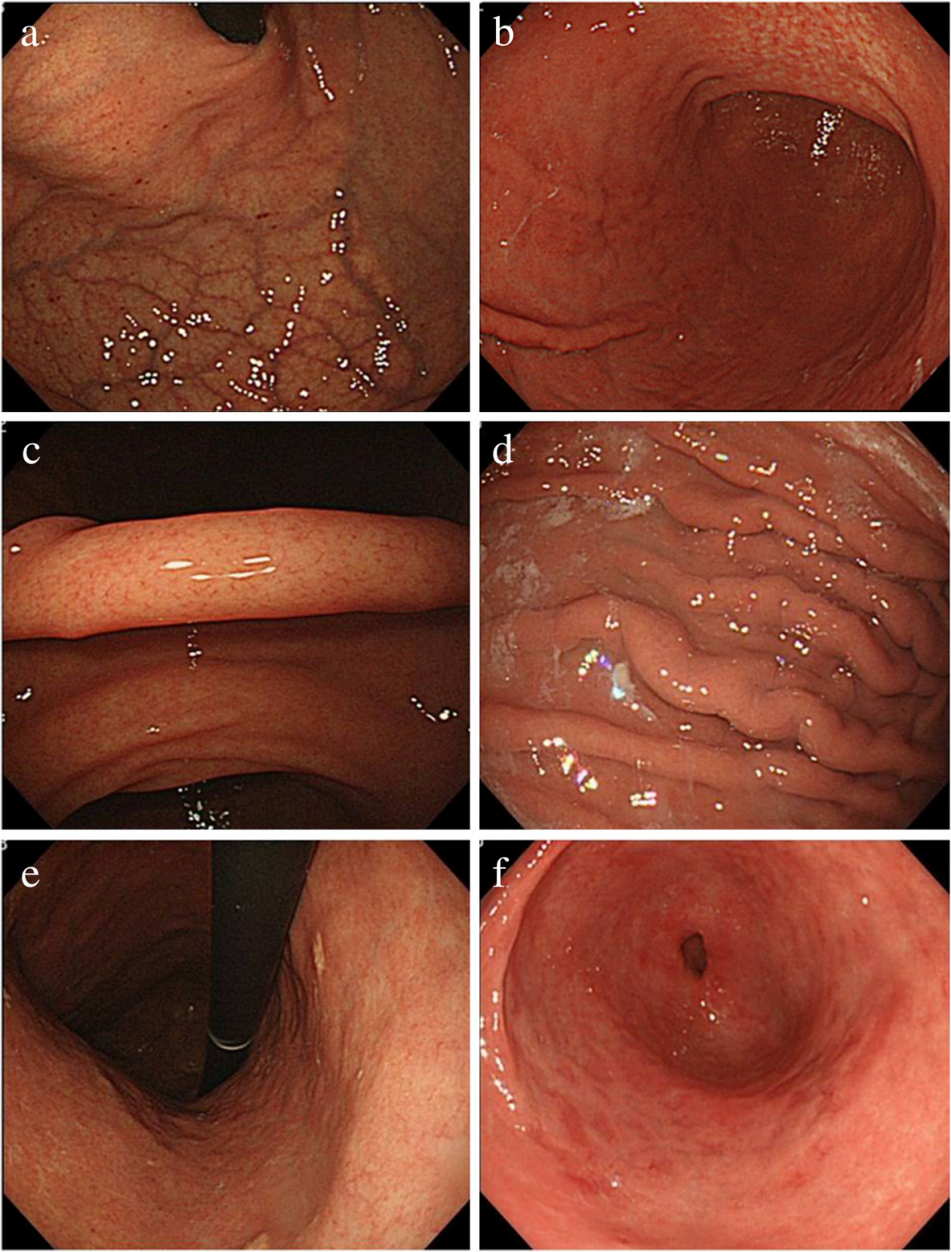
**Different sites of the stomach showing *H. pylori *infection status. a**. Cardia with hemorrhage. **b**. Lesser and greater curvature of lower body with atrophy, and spotty erythema. **c**. Angulus with regular arrangement of collecting venules. **d**. Greater curvature of the upper body with exudates, edema, and rugal hyperplasia. **e**. Lesser curvature of lower body with xanthoma. **f**. Antrum with motteled patchy erythema.

We routinely record about 50–60 images at fixed sites of the esophagus, stomach, and duodenum in all cases and save them to the electronic endoscopic database (Solemio ENDO, Olympus Medical Systems). We selected six photos of specific sites of the antrum, angulus, lesser and greater curvature of the lower body, greater curvature of the upper body, and cardia of the stomach (Figure 1) from the electronic endoscopic database in each case, and endoscopic findings were then evaluated.

The following 11 distinctive endoscopic findings related to *H. pylori* infection status (uninfected, infected, and eradicated) were used for analysis: regular arrangement of collecting venules (RAC) [16], atrophic change [17,18], rugal hyperplasia [19], edema [20], spotty erythema [20], linear erythema [20], hemorrhage [20], exudate [20], fundic gland polyp [21], xanthoma [22], and motteled patchy erythema (MPE) [23]. Before judging, we held several seminars to obtain a consensus on the relation between *H. pylori* infection status and distinctive endoscopic findings [16-23] using typical images selected from the electronic endoscopic database. We then judged *H. pylori* infection status and categorized it as *H. pylori*-uninfected, *H. pylori*-infected, or *H. pylori*-eradicated on the basis of endoscopic findings. Endoscopic images were assessed by six endoscopists who were grouped according to endoscopic experience as follows: two beginner (<500 upper endoscopies), two intermediate (1500–5000 upper endoscopies), and two advanced (>5000 upper endoscopies). All six endoscopists were blinded to the clinical information of examined cases.

### Training for endoscopic assessment of H. pylori infection status

Training of beginners was conducted in a systematical manner over two years of daily clinical practice and entailed the following: 1) recording the presence or absence of the 11 distinctive endoscopic findings into the electronic endoscopic database for all patients; 2) recording the prediction of *H. pylori* infection status on the basis of endoscopic findings. Two years after initial diagnosis, diagnosis of *H. pylori* infection status and endoscopic findings were reassessed in the same manner for all cases.

### Statistical analysis

Diagnostic yield was calculated as a positive predictive value using the results of the endoscopic evaluation of 77 cases. To calculate the diagnostic yield of all six physicians, the results of the 77 cases were totaled, and 462 cases were used for analysis. Diagnostic yield was then compared among endoscopists with different levels of experience using the Chi-squared test. We calculated the value of intra- and inter-observer agreement using the kappa statistic [24] to clarify the reproducibility of the endoscopic diagnosis of *H. pylori* infection status. Kappa values (K) >0.80 denoted excellent, >0.60–0.80 good, >0.40–0.60 moderate, >0.20–0.40 fair, and ≤0.20 poor [24].

To determine intra-observer agreement, all six physicians reassessed the same endoscopic images one week after the first evaluation in a different order. Inter-observer agreement among all six endoscopists and between endoscopist pairs of different levels of experience (two each for beginner, intermediate, and advanced) was calculated. Inter-observer agreement of the 11 endoscopic findings was calculated in the same manner. After training the beginners for two years, diagnostic yield and inter-observer agreement were reassessed and compared against the values of the initial diagnosis using the McNemar test.

Values of p < 0.05 were considered significant. All statistical analysis was performed using Stata version 10 software (StataCorp, Lakeway Drive College Station, TX).

## Results

### Patient characteristics

A total of 77 patients (32 men and 45 women; mean age (SD), 39.7 (13.4) years) and 462 images were assessed. Of them, 28 were *H. pylori*-uninfected, 28 were -infected, and 21 were -eradicated. Of the 21 eradicated cases, 18 had information on the date of eradication therapy. The mean (SD) period from eradication therapy to endoscopy of these 18 cases was 28 (32) months.

### Diagnostic yield of endoscopic diagnosis of H. pylori infection status

The yield of endoscopic diagnosis of *H. pylori* infection status is shown in Table 1. The yield was highest for *H. pylori*-uninfected (88.9%), followed by *H. pylori*-infected (62.1%), and *H. pylori*-eradicated (55.8%) for all six endoscopists. The same order of yield for *H. pylori* infection status was also found within each level of endoscopic experience: the yield of *H. pylori*-infected and -eradicated was lower in the beginner group than in the intermediate and advanced groups, but differences were not statistically significant (p > 0.05).

**Table 1:** **Diagnostic yield of *H. pylori *infection status on endoscopy**

	***H. pylori*-uninfected**	***H. pylori*-infected**	***H. pylori*-eradicated**
All physicians* (n = 6)	88.9% (82.3–93.6%)	62.1% (55.3–68.7%)	55.8% (46.1–65.1%)
Individual physicians**			
Beginner 1	82.6% (61.2–95.0%)	55.3% (38.3–71.4%)	37.5% (15.2–64.6%)
Beginner 2	90.9% (70.8–98.9%)	54.8% (36.0–72.7%)	50.0% (29.1–70.9%)
Intermediate 1	91.3% (72.0–98.9%)	67.6% (49.5–82.6%)	65.0% (40.8–84.6%)
Intermediate 2	82.6% (61.2–95.0%)	66.7% (49.0–81.4%)	66.7% (49.0–81.4%)
Advanced 1	90.5% (69.6–98.8%)	63.9% (46.2–79.2%)	50.0% (27.2–72.8%)
Advanced 2	95.7% (78.1–99.9%)	64.1% (47.2–78.8%)	80.0% (51.9–95.7%)

^*^Analyzed cases: n = 462. ^**^Analyzed cases: n = 77. 95% confidence interval values are given in parentheses.

Abbreviation: *H. pylori, Helicobacter pylori.*

### Intra- and inter-observer agreement of endoscopic diagnosis of H. pylori infection status

Intra-observer agreement of all six physicians was relatively good (K > 0.6) irrespective of endoscopic experience (Table 2). Inter-observer agreement was moderate (K = 0.46) in the beginner group but high in the intermediate and advanced groups (K > 0.6) (Table 2).

**Table 2:** **Intra- and inter-observer agreement of *H. pylori *infection status on endoscopy**

	**Intra-observer agreement**	**Inter-observer agreement**
Beginner 1	0.65	0.46
Beginner 2	0.62	
Intermediate 1	0.72	0.78
Intermediate 2	0.74	
Advanced 1	0.67	0.65
Advanced 2	0.82	

Note: All values calculated using kappa statistics.

### Inter-observer agreement of endoscopic findings associated with H. pylori infection status

The inter-observer agreement of the endoscopic findings is shown in Table 3. A high agreement among the six endoscopists was found for atrophic change (K = 0.63), hemorrhage (K = 0.62), and RAC (K = 0.63). Of the different levels of endoscopic experience, beginners showed the lowest inter-observer agreement for all findings, except for exudate and xanthoma.

**Table 3:** Inter-observer agreement of endoscopic findings

	**All physicians**	**Beginner**	**Intermediate**	**Advanced**	**Beginner (two years later)**
Atrophic change	0.69	0.54	0.75	0.81	0.77
RAC	0.63	0.58	0.87	0.50	0.81
Hemorrhage	0.62	0.29	0.77	0.81	0.64
Fundic gland polyp	0.55	0.17	0.58	0.67	0.75
Rugal hyperplasia	0.51	0.42	0.65	0.54	0.84
Spotty erythema	0.51	0.53	0.54	0.72	0.57
Linear erythema	0.51	0.15	0.80	0.72	0.55
Exudate	0.48	0.51	0.58	0.38	0.59
MPE	0.48	0.27	0.67	0.47	0.63
Edema	0.46	0.27	0.58	0.53	0.38
Xanthoma	0.35	0.22	0.06	0.75	0.55

Note: All values calculated using kappa statistics.

### Change in endoscopic diagnostic yield and endoscopic findings after two years of training

**Figure 2:**
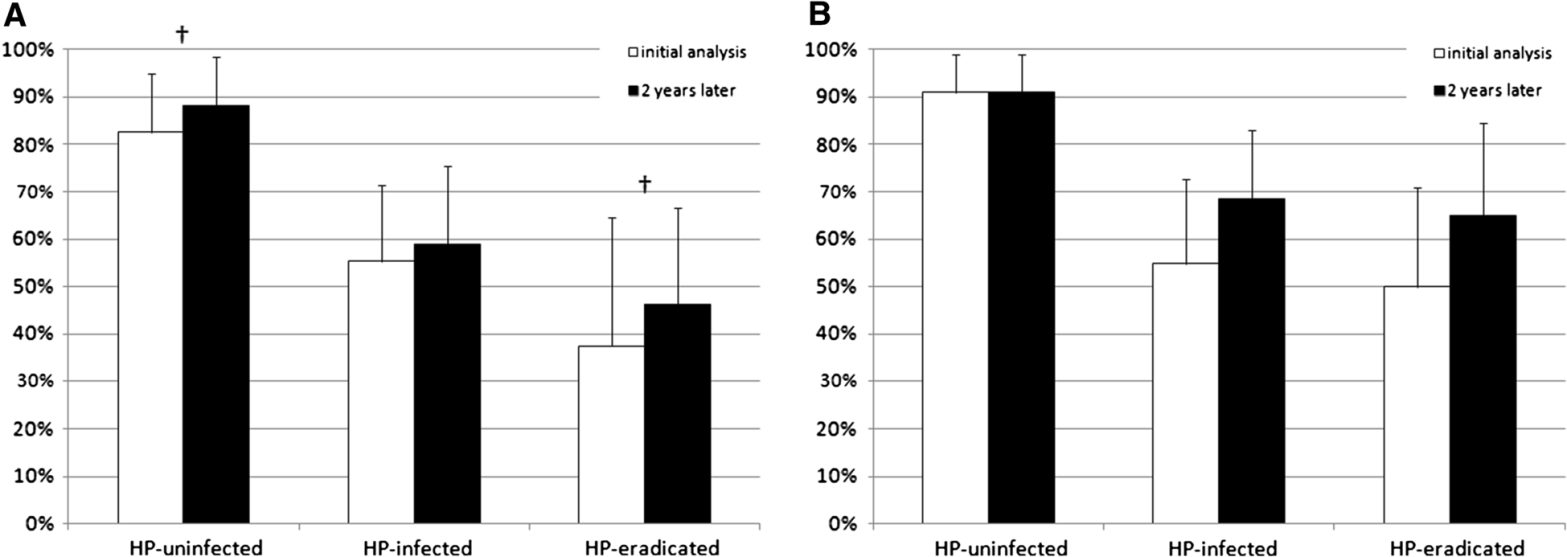
**Comparison of diagnostic yield of *H. pylori *infection status in two beginner endoscopists before and after training.** Diagnostic yield of Beginner 1 **(A)** and Beginner 2 **(B)**. ^†^P-values are statistically significant at <0.05. Analysis performed using Mcnemer’s test. Error bar represents the 95% CI of diagnostic yield. Abbreviations: HP, *H. pylori;* PPV, positive predictive value.

Figure 2 shows the diagnostic yield of all cases of *H. pylori* infection status on endoscopy before and after training. A significant increase was noted in diagnostic yield of *H. pylori*-uninfected (82.6% and 88.6%, p < 0.05) and *H. pylori*-eradicated (37.5% and 46.2%, p < 0.05) in beginner 1. Slight increases were also evident for the diagnostic yield of *H. pylori*-infected (55.3% and 58.6%, P = 0.41) in beginner 1, *H. pylori*-infected (54.8% and 68.6%, P = 0.25) in beginner 2, and *H. pylori*-eradicated (50.0% and 65.0%, P = 0.25) in beginner 2. In contrast, the diagnostic yield of *H. pylori*-uninfected in beginner 2 remained high (90.0% and 90.9%) after training.

## Discussion

In previous studies on endoscopic diagnosis for *H. pylori* infection, one or two tests were regarded as the gold standard. However accurate diagnosis of *H. pylori* infection is difficult; thus combining several diagnostic approaches is preferable [25,26]. In our study, we used histology, serology, and the urea breast test to diagnose *H. pylori* infection status accurately, making the results highly reliable.

The low diagnostic yield for *H. pylori*-eradicated cases (55.8%) may reflect insufficient knowledge of typical endoscopic images of *H. pylori*-eradicated mucosa because few studies have reported such endoscopic findings [19,27]. However, this value is still considered relatively good, because it is not easy to discriminate between *H. pylori*-eradicated and -uninfected cases, even when using serological or UBT testing [28,29]. *H. pylori* eradication is currently recommended worldwide, so the number of eradicated patients is expected to increase. However, gastric cancer is sometimes detected on endoscopy even after eradication [3,4], therefore, it is important that *H. pylori*-eradicated cases can be endoscopically distinguished from *H. pylori*-uninfected ones.

Although a number of endoscopic studies on *H. pylori* infection (uninfected versus infected) have been reported, results have been contradictory. Khaloo et al. evaluated the updated Sydney system (USS) on endoscopy and obtained a low diagnostic yield of 41.8% [8], and Belair et al. concluded that endoscopic diagnosis of *H. pylori* infection is not useful because of a low ROC of 0.55 [9]. Redeen et al. also reported a low diagnostic yield (43–53%) [10], while a slightly higher diagnostic yield of 64% was obtained by Bah et al. [7].

In contrast, a study of *H*. *pylori* infection in highly endemic areas by Mihara et al. obtained a high diagnostic yield of 79.5% in accordance with the USS [11]. The inconsistency in diagnostic yields might have been caused by differences in regional disease prevalence. In the present study, diagnostic yield was relatively low compared with previous studies, as reflected by the difficulty in distinguishing between *H. pylori*-infected and *H. pylori*-eradicated cases.

The diagnostic yield in *H. pylori*-uninfected cases was high. We believe this is because many of the endoscopists correctly identified RAC [16], hemorrhage [20], and fundic gland polyps [21], all of which are characteristic of *H. pylori-*uninfected mucosa. These findings in fact attained good inter-observer agreement.

We hypothesized that diagnostic yield is influenced by endoscopist experience and found that scores for *H. pylori*-infected and -eradicated cases were low for beginners compared with intermediate and advanced endoscopists. In addition, inter-observer agreement on *H. pylori* infection status between beginners was noticeably inconsistent, presumably because endoscopic findings by beginners varied among cases compared with intermediate and advanced endoscopists. In intermediate and advanced endoscopists, the definition of endoscopic findings appears to have been well established, and thus the extraction was consistent.

Lastly, the diagnostic yield for *H. pylori*-infected and -eradicated gastric mucosa was increased in the two beginners after two years of training, and subsequently, their post-training inter-observer agreement has also improved. We believe that training improved their understanding of individual endoscopic findings that defined different states of *H. pylori* infection, resulting in consistent inter-observer agreement and higher diagnostic yield.

Because no video-based evaluation was performed, we could not observe detailed mucosal patterns and some local findings that may have been present, which is a limitation of our study. Endoscopists may not spend sufficient time performing detailed observation of the gastric mucosa in *H. pylori*-uninfected cases, whereas examination of *H. pylori*-infected gastric mucosa with inflammation is expected to be time-consuming and detailed. Therefore, we believe that blindness is maintained better with the use of photographic images.

Our diagnostic yield of approximately 90% for *H. pylori*-uninfected cases suggests that patients with a low risk of gastric cancer can be determined through screening endoscopy. Although, the diagnostic yield for *H. pylori*-infected and -eradicated cases was lower than that for uninfected cases, more than half of the patients who underwent screening endoscopy were determined to be at high risk of gastric cancer, so we performed careful observation of the gastric mucosa during endoscopy. In addition, we recommend additional tests such as a UBT or serology test for these patients. This diagnostic strategy may be efficient and cost-effective for the early detection of gastric cancer, which is an important clinical aspect of this study. Only a few studies have reported training methods to improve endoscopic diagnostic fields [30,31]. The results of this study suggest the importance of clearly defining disease-associated endoscopic findings and training endoscopists to pay attention to and extract these findings in daily clinical practice.

## Conclusions

In conclusion, this study revealed that the diagnostic yield for *H. pylori* infection status was highest for *H. pylori*-uninfected, followed by -infected, and -eradicated cases. Accuracy was low in beginners but improved after two years of training. Extraction of endoscopic findings for the diagnosis of *H. pylori* infection status appears to be useful, and the beneficial effects can be enhanced by training.

## Abbreviations

H. pylori: Helicobacter pylori; UBT: Urea breath test; RAC: Regular arrangement of collecting venules; MPE: Mottled patchy erythema; PPV: Positive predictive value; K: Kappa; USS: Updated Sydney system.

## Competing interests

The authors declare that they have no competing interests.

## Authors’ contributions

KW participated in data acquisition and interpretation and wrote the manuscript. NN participated in the design of the study, data interpretation, performed endoscopy, and contributed to the writing of the manuscript. TS participated in the design of the study and contributed to statistical analysis. RN helped with data acquisition. EF, TS, NA, CY, and MK performed endoscopic assessment. JA and NU advised on the design of the study and contributed to the writing of the manuscript. MM advised on the content of the revised manuscript and contributed to the writing of the revised manuscript. All authors discussed the content and commented on the manuscript. All authors read and approved the final manuscript.

## Pre-publication history

The pre-publication history for this paper can be accessed here:

http://www.biomedcentral.com/1471-230X/13/128/prepub
